# HER2 alterations across solid tumors: implications for comprehensive testing

**DOI:** 10.1093/oncolo/oyaf258

**Published:** 2025-08-19

**Authors:** Ahmed Ismail, Chinmay T Jani, Nusrat Jahan, Malla Midhun, Arnab Basu, Garima Gupta, Bassel El-Rayes, Sejong Bae, Tyler Mattox, Cyntanna Hawkins, Rebecca C Arend, Mehmet Akce, Yanis Boumber, Aakash Desai

**Affiliations:** Section of Hematology & Oncology, Department of Medicine, The University of Alabama at Birmingham and O’Neal Comprehensive Cancer Center, Birmingham, AL 35233, United States; Department of Medicine, Eastern Virginia Medical School at Old Dominion University, Norfolk, VA 23507, United States; Division of Oncology, Department of Medicine, University of Miami Sylvester Comprehensive Cancer Center, Miami, FL 33136, United States; Section of Hematology & Oncology, Department of Medicine, The University of Alabama at Birmingham and O’Neal Comprehensive Cancer Center, Birmingham, AL 35233, United States; Section of Hematology & Oncology, Department of Medicine, The University of Alabama at Birmingham and O’Neal Comprehensive Cancer Center, Birmingham, AL 35233, United States; Section of Hematology & Oncology, Department of Medicine, The University of Alabama at Birmingham and O’Neal Comprehensive Cancer Center, Birmingham, AL 35233, United States; Section of Hematology & Oncology, Department of Medicine, The University of Alabama at Birmingham and O’Neal Comprehensive Cancer Center, Birmingham, AL 35233, United States; Section of Hematology & Oncology, Department of Medicine, The University of Alabama at Birmingham and O’Neal Comprehensive Cancer Center, Birmingham, AL 35233, United States; Division of General Internal Medicine and Population Science, Department of Medicine and O’Neal Comprehensive Cancer Center, The University of Alabama at Birmingham, Birmingham, AL 35233, United States; Caris Life Sciences, Phoenix, AZ 85040, United States; Caris Life Sciences, Phoenix, AZ 85040, United States; Division of Gynecologic Oncology, Department of OBGYN, The University of Alabama at Birmingham and O’Neal Comprehensive Cancer Center, Birmingham, AL 35233, United States; Section of Hematology & Oncology, Department of Medicine, The University of Alabama at Birmingham and O’Neal Comprehensive Cancer Center, Birmingham, AL 35233, United States; Section of Hematology & Oncology, Department of Medicine, The University of Alabama at Birmingham and O’Neal Comprehensive Cancer Center, Birmingham, AL 35233, United States; Section of Hematology & Oncology, Department of Medicine, The University of Alabama at Birmingham and O’Neal Comprehensive Cancer Center, Birmingham, AL 35233, United States

**Keywords:** HER2, ERBB2, HER2 overexpression, immunohistochemistry, copy number variation, HER2 mutational status

## Abstract

**Purpose:**

ERBB2 (HER2) alterations (eg, overexpression, amplification, and mutations) are known to drive tumor progression. These changes, particularly in non-breast and gastric/gastroesophageal cancers, remain poorly characterized. With pan-tumor approval of HER2-targeted therapies like Trastuzumab deruxetecan (T-DXd), understanding ERBB2 alterations across diverse cancers is crucial.

**Methods:**

HER2 analysis was conducted on 653 solid tumor specimens at the University of Alabama, using immunohistochemistry (IHC), copy number (CN) variation (CNV) assessment, and mutational profiling. The correlation between CN amplification and IHC expression was evaluated using Somers’ *D* ordinal association.

**Results:**

Of the 653 cases, HER2 IHC scores were distributed as 3+ (3.1%), 2+ (13.2%), and 1+ (19.8%), with 63.9% being IHC-negative. ERBB2 CN amplification was observed in 3.1%, with 75% exhibiting IHC3+. Pathogenic mutations were found in 3.1%, with low IHC3+ rates (5%). Among samples with ERBB2 mutations, only 3 had CN amplifications (1-positive, 2-intermediate). Somers’-*D* analysis revealed a strong association between CNV and IHC expression (*D* = 0.73, *P* < .001).

**Conclusion:**

This study highlights ERBB2 alterations across diverse cancers, demonstrating their heterogeneity and clinical significance. ERBB2 mutation-carrying tumors are less likely to have HER2 protein 3+ expression or CN amplification, indicating the need for comprehensive genomic analysis to identify those patients. In the context of pan-tumor approval of T-DXd for HER2, findings support integrating genomic and phenotypic data to enhance diagnostic precision and inform therapeutic decision-making. Comprehensive ERBB2 (HER2) testing across tumor types is essential to expand access to HER2-targeted therapies.

Implications for Practice *Key objective:* To highlight the clinical significance of comprehensive ERBB2 (HER2) testing in identifying eligible patients for HER2-targeted therapies, particularly following the FDA’s accelerated approval of T-DXd for adult patients with metastatic HER2-positive (IHC 3+) relapsed/refractory solid tumors on April 5, 2024. *Knowledge generated:* Comprehensive ERBB2 (HER2) testing is vital for identifying patients for HER2-targeted therapies and guiding treatment decisions, as HER2 positivity can be detected in about 36% of various solid tumors. Unlike ERBB2 mutations, ERBB2 copy number amplification is strongly associated with HER2 expression and higher levels of IHC results.

## Introduction

Human epidermal growth factor receptor 2 (HER2) is a transmembrane growth factor receptor with intrinsic tyrosine kinase activity, encoded by ERBB2 gene, and plays a critical role in cell growth, survival, and proliferation. Notably, ERBB2 (*HER2)* can act as an oncogenic driver, and aberrant HER2 activation can lead to unregulated cell growth, survival, and proliferation.^1^

Alternations of ERBB2, including overexpression and mutation, have been observed in multiple tumor types. HER2 overexpression by immunohistochemistry (IHC) is observed in approximately 15%-30% of breast cancers and 23% of gastric/gastroesophageal junction carcinomas.^2–4^ Other epithelial-origin tumors, including colorectal cancer (CRC), esophageal, bladder, cholangiocarcinoma, gallbladder, cervical, and endometrial cancers, also demonstrate HER2 IHC overexpression.^5–7^ Furthermore, ERBB2 mutations have been detected across various cancer types, with the highest prevalence reported in bladder cancer (9%-18%), followed by uterine and cervical cancers (6%), CRC (5.8%), lung (4%), and breast cancer (4%).^8–11^ Additionally, a comprehensive analysis of 111,176 tumors found that 3.5% harbored ERBB2 mutations.

Certain ERBB2 mutations confer resistance to targeted therapies.^12^ For instance, exon 20 insertions in non-small-cell lung cancer (NSCLC) promote continuous HER2 heterodimerization and activation, even in ligand binding absence.^13^^,^^14^ Similarly, ERBB2 gene amplification throughout the tumor may confers resistance to EGFR-targeted monoclonal antibodies in CRC.^15^

Given its pivotal role in cancer progression, ERBB2 (HER2) has become a critical target for cancer therapy. The development of trastuzumab, an anti-HER2 monoclonal antibody, revolutionized cancer therapeutics. More recently, the antibody-drug conjugate (ADC) fam-trastuzumab deruxtecan-nxki (T-DXd) has significantly improved outcomes in ERBB2/HER2-overexpressing and mutant cancers.^16^ In April 2024, T-DXd received accelerated approval for adult patients with unresectable or metastatic HER2-positive (IHC3+) solid tumors who have received prior systemic treatment and have no satisfactory alternative treatment options.^17^ ADCs consist of a humanized monoclonal antibody (predominantly IgG) conjugated to a cytotoxic payload via a molecular linker, delivering highly potent cytotoxicity to cancer cells while minimizing systemic toxicity.^18^ Despite the promising results of trials,^16^^,^^17^ a significant challenge remains in identifying HER2-positive tumors among tumor types that lack routine ERBB2 (HER2) testing. Moreover, the real-world applicability and utilization of T-DXd require further exploration. To address such gaps, we present a unique dataset from our institution that includes ERBB2 (HER2) testing results, encompassing IHC expression, copy number variation, and mutational status. This analysis provides valuable insights into HER2’s clinical significance and the potential utility of T-DXd in diverse patient populations.

## Methods

Solid tumor specimens from patients treated at the University of Alabama (UAB), O’Neal Comprehensive Cancer Center and submitted to Caris Life Sciences Molecular Profiling Laboratory in Arizona between May 2021 and March 2024 were evaluated for ERBB2 (HER2) analysis. Human investigations were performed after approval by a local Human Investigations Committee and in accord with an assurance filed with and approved by the Department of Health and Human Services, where appropriate. Data were anonymized to protect the identities of individuals involved in the research. Informed consent was also obtained from participants or guardians.

Molecular profiling was conducted at Caris Life Sciences, a CAP/CLIA-certified lab. FFPE tumor samples underwent H&E review by a board-certified pathologist, with tumor enrichment achieved via manual microdissection. Minimum tumor content and dissection area thresholds varied by assay type (eg, ≥20% tumor nuclei and ≥20–25 mm^2^ for WES or MI Cancer Seek). DNA and RNA profiling evolved over time: initially performed with a 592-gene panel (NextSeq), later upgraded to whole-exome sequencing (WES) and whole transcriptome sequencing (WTS) using the NovaSeq 6000 platform. In 2023, profiling transitioned to the MI Tumor Seek Hybrid platform, which extracts and analyzes total nucleic acids simultaneously. Bioinformatics pipelines included BWA, STAR, SamTools, Salmon, and others, with alignment to hg19 or hg38 genomes. Variants were interpreted using American College of Medical Genetics (ACMG) guidelines and classified accordingly. Only pathogenic or likely pathogenic variants were reported, with germline variants filtered out using established databases such as dbSNP.

HER2 copy number (CN) amplification was assessed by comparing the average sequencing depth of each exon in the sample to a pre-calibrated reference value. Positive amplification was defined as CN > 6. Intermediate amplification was defined when the CN ≥ 6; however, the statistical confidence in the measurement does not meet the predefined threshold required to conclusively categorize the result as true amplification. Unlike positive amplification, intermediate results suggest variable or lower IHC expression due to biological heterogeneity or technical limitations and necessitate further investigation or verification using additional methods.

ERBB2 variants (single-nucleotide variants [SNVs] and insertions/deletions [INDELs]) were classified according to the ACMG standards. IHC assays were performed using FDA-approved companion diagnostic or FDA-cleared tests, following the manufacturer’s instructions, specifically the HER2/neu (PATHWAY anti-HER-2/neu [4B5], VENTANA^19^). Our study assessed HER2 status across various tumor types using IHC. For non-breast tumors, we applied the American Society of Clinical Oncology/College of American Pathologists (ASCO/CAP) gastric cancer scoring criteria. For breast cancer specimens, we adhered to the ASCO/CAP breast cancer guidelines.

The scoring criteria utilized were as follows:

*Gastric cancer HER2 IHC scoring:*

0/1+ (Negative): Cancer cell cluster with a faint or barely perceptible membranous reactivity irrespective of percentage of positive tumor cells.

2+ (Equivocal): Cancer cell cluster with a weak to moderate complete, basolateral, or lateral membranous reactivity irrespective of percentage of positive tumor cells.

3+ (Positive): Cancer cell cluster with a strong complete basolateral, or lateral membranous reactivity irrespective of percentage of positive tumor cells.

*Breast cancer HER2 IHC scoring:*

0: No staining or incomplete membrane staining in <10% of tumor cells.

1+: Faint/barely perceptible incomplete membrane staining in >10% of tumor cells.

2+: Weak to moderate complete membrane staining in >10% of tumor cells.

3+: Uniform, intense circumferential membrane staining in >10% of tumor cells.

Data from the UAB patients’ test reports were extracted and analyzed using descriptive statistics. The correlation between ERBB2 (HER2) CNV and IHC expression was assessed. Somers’ *D*, an ordinal association measure, was employed to evaluate the strength and direction of the relationship between CNV (independent variable) and IHC expression (dependent variable). This analysis provided insights into the extent to which CN amplification predicts HER2 protein expression levels, facilitating a deeper understanding of the concordance between genomic alterations and phenotypic expression in solid tumors. Results were reported with corresponding confidence intervals and *P*-values to ensure statistical rigor.

## Results

### Overview of tumor types and HER2 expression

**Figure 1.**
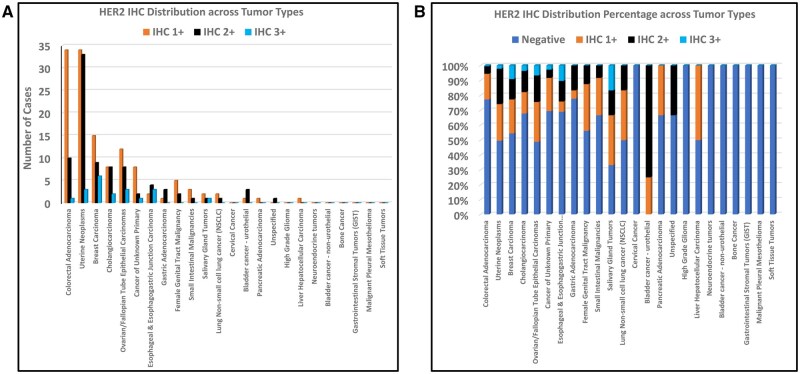
Distribution of immunohistochemistry results across different tumor types.

We analyzed 653 tumor cases across 24 distinct tumor types (Figure 1A, Table 1). The most represented tumors included colorectal adenocarcinoma (30.3%, *n* = 198), uterine neoplasms (21.3%, *n* = 139), metastatic breast carcinoma (10.1%, *n* = 66), cholangiocarcinoma (8.6%, *n* = 56), and epithelial ovarian/fallopian tube carcinomas (6.9%, *n* = 45). The remaining tumors collectively accounted for 22.8% (*n* = 149) of cases.

**Table 1.** Samples included in our cohort.

Tumor type	Total number of cases	Percentage of the entire cohort	Number of cases with any detected pathogenic alteration	Alteration %	HER2 IHC 3+	IHC +3 %	HER2 IHC 2+	IHC 2+ %	HER2 IHC 1+	IHC 1+ %	CNV amplification +ve	CNV +ve %	CNV amplification intermediate	CNV intermediate %	Positive mutation	Positive mutation %	VUS mutation
**Colorectal adenocarcinoma**	198	30.3	48	24.2	1	0.5	10	5.1	34	17.2	1	0.5	4	2.0	5	2.5	5
**Uterine neoplasms**	139	21.3	71	51.1	3	2.2	33	23.7	34	24.5	3	2.2	4	2.9	6	4.3	6
**Breast carcinoma**	66	10.1	32	48.5	6	9.1	9	13.6	15	22.7	5	7.6	1	1.5	4	6.1	1
**Cholangiocarcinoma**	56	8.6	19	33.9	2	3.6	8	14.3	8	14.3	3	5.4	5	8.9	0	0.0	0
**Epithelial ovarian/Fallopian tube carcinomas**	45	6.9	23	51.1	3	6.7	8	17.8	12	26.7	2	4.4	3	6.7	1	2.2	0
**Cancer of unknown primary**	36	5.5	11	30.6	1	2.8	2	5.6	8	22.2	1	2.8	0	0.0	1	2.8	2
**Esophageal/Esophagogastric junction carcinoma**	29	4.4	10	34.5	3	10.3	4	13.8	2	6.9	4	13.8	2	6.9	1	3.4	1
**Gastric adenocarcinoma**	18	2.8	4	22.2	0	0.0	3	16.7	1	5.6	0	0.0	0	0.0	0	0.0	0
**Female genital tract malignancy**	16	2.5	8	50.0	0	0.0	2	12.5	5	31.3	0	0.0	1	6.3	0	0.0	0
**Small intestinal malignancies**	12	1.8	5	41.7	0	0.0	1	8.3	3	25.0	0	0.0	0	0.0	1	8.3	0
**Salivary gland tumors**	6	0.9	4	66.7	1	16.7	1	16.7	2	33.3	1	16.7	0	0.0	0	0.0	0
**Lung non-small-cell lung cancer**	6	0.9	3	50.0	0	0.0	1	16.7	2	33.3	0	0.0	0	0.0	0	0.0	0
**Cervical cancer**	5	0.8	1	20.0	0	0.0	0	0.0	0	0.0	0	0.0	0	0.0	1	20.0	0
**Bladder cancer—urothelial**	4	0.6	4	100.0	0	0.0	3	75.0	1	25.0	0	0.0	1	25.0	0	0.0	0
**Pancreatic adenocarcinoma**	3	0.5	1	33.3	0	0.0	0	0.0	1	33.3	0	0.0	0	0.0	0	0.0	0
**Unspecified**	3	0.5	1	33.3	0	0.0	1	33.3	0	0.0	0	0.0	0	0.0	0	0.0	0
**High-grade glioma**	2	0.3	1	50.0	0	0.0	0	0.0	0	0.0	0	0.0	1	50.0	0	0.0	0
**Liver hepatocellular carcinoma**	2	0.3	1	50.0	0	0.0	0	0.0	1	50.0	0	0.0	0	0.0	0	0.0	0
**Neuroendocrine tumors**	2	0.3	0	0.0	0	0.0	0	0.0	0	0.0	0	0.0	0	0.0	0	0.0	0
**Bladder cancer—non-urothelial**	1	0.2	0	0.0	0	0.0	0	0.0	0	0.0	0	0.0	0	0.0	0	0.0	0
**Bone cancer**	1	0.2	0	0.0	0	0.0	0	0.0	0	0.0	0	0.0	0	0.0	0	0.0	0
**Gastrointestinal stromal tumors**	1	0.2	1	100.0	0	0.0	0	0.0	0	0.0	0	0.0	1	100.0	0	0.0	0
**Malignant pleural mesothelioma**	1	0.2	0	0.0	0	0.0	0	0.0	0	0.0	0	0.0	0	0.0	0	0.0	0
**Soft tissue tumors**	1	0.2	0	0.0	0	0.0	0	0.0	0	0.0	0	0.0	0	0.0	0	0.0	0
**Results for all tumor types**	**653**	**100**	**248**	**38.0**	**20**	**3.1**	**86**	**13.2**	**129**	**19.8**	**20**	**3.1**	**23**	**3.5**	**20**	**3.1**	**15**

Given the clinical relevance of tumors with HER2 IHC3+ expression, we further evaluated the prevalence of HER2 IHC overexpression (1+, 2+, and 3+), using ASCO/CAP breast cancer guidelines for breast cancer specimens and ASCO/CAP gastric cancer scoring criteria for non-breast cancers. The highest proportion of HER2 IHC 3+ expression was observed in salivary gland tumors (16.7%, *n* = 1), followed by esophageal/esophagogastric junction carcinoma (10.3%, *n* = 3), metastatic breast carcinoma (9.1%, *n* = 6), epithelial ovarian/fallopian tube carcinomas (6.7%, *n* = 3), and cholangiocarcinoma (3.6%, *n* = 2). Across all analyzed tumor types, HER2 IHC scores were distributed as follows: 3.1% (*n* = 20) scored IHC3+, 13.2% (*n* = 86) scored IHC2+, and 19.8% (*n* = 129) scored IHC1+. The remaining majority were IHC-negative (63.9%, *n* = 418) (Figure 1B).

### ERBB2 CN amplification and mutational analysis

**Figure 2.**
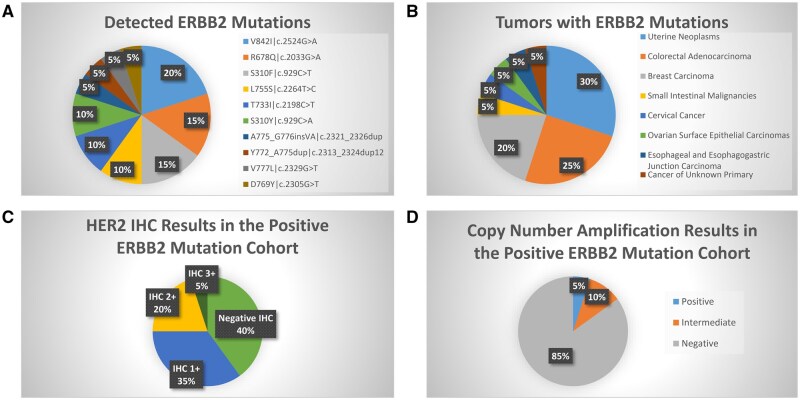
Detected ERBB2 mutations.

ERBB2 CN amplification analysis revealed that 3.1% (*n* = 20) of cases exhibited positive amplification, while 3.5% (*n* = 23) displayed intermediate amplification levels. A 3.1% (*n* = 20) of cases harbored pathogenic ERBB2 mutations, and an additional 2.3% (*n* = 15) had VUS (Figure 2A-D). The most prevalent pathogenic mutations identified were V842I (c.2524G>A), R678Q (c.2033G>A), S310F (c.929C>T), and L7555 (c.2264T>C), which together accounted for 60% of all detected mutations. Other mutations included T733 (c.2198C>T), S310 (c.929C>A), A775_G776insV (c.2321_2326dup), Y772_A775dup (c.2313_2324dup12), V777L (c.2329G>T), and D769Y (c.2305G>T) (Figure 2A). Among the 20 patients with pathogenic ERBB2 mutations, only one patient exhibited positive CN amplification [A775_G776insVA (c.2321_2326dup) in breast cancer]. Additionally, intermediate CN amplification was observed in 2 patients: one with T733I (c.2198C>T) in esophageal cancer and another with S310Y (c.929C>A) in ovarian cancer. Overall, no significant association was found between CN amplification and mutation analysis, further emphasizing the distinct and independent nature of these HER2 alterations.

### Correlation between ERBB2 CN amplification and HER2 IHC expression

In the positive CN amplification group (3.1%, *n* = 20), 75% of cases exhibited IHC3+ expression, 20% had IHC2+ expression, and 5% were IHC1+. In contrast, the intermediate CN amplification group (3.5%, *n* = 23) showed 8.7% IHC3+ expression, 47.8% IHC2+ expression, and 21.7% IHC1+. For tumors with pathogenic ERBB2 mutations, the HER2 IHC3+ expression rate was lower, observed in only 5% of cases, while IHC2+ and IHC1+ expression were observed in 20% and 35%, respectively (Figure 2C-D). The correlation between ERBB2 CN amplification and HER2 IHC expression was analyzed using Somers’ *D*, an ordinal association measure, showing a value of 0.73 (95%CI: 0.607, 0.854) and a strong positive association. This statistically significant correlation (*P* < .001) demonstrates that increased ERBB2 CN amplification is associated with higher HER2 IHC expression levels.

## Discussion

ERBB2 (HER2) aberrations, including amplification, overexpression, and activating mutations, are well-established crucial oncogenic drivers in various cancer types. These alterations promote tumor growth and progression by activating downstream signaling pathways. While ERBB2/HER2 amplification and overexpression have been extensively studied in breast and gastric cancers, where they are associated with poor prognosis and aggressive tumor biology,^20^^,^^21^ their prevalence, prognostic significance, and therapeutic implications in other tumor types remain less well-defined. Additionally, the relationship between CNV, IHC expression, and mutational status has not been comprehensively explored.

To address this gap, our study analyzed 653 tumor samples spanning 24 distinct tumor types, focusing on ERBB2 (HER2) expression, amplification, and mutational patterns. A key finding of our study was the strong positive correlation between ERBB2 CN amplification and HER2 IHC expression, with a Somers’ *D* value of 0.73 (95% CI: 0.607, 0.854; *P* < .001), which is consistent with recent reported data in the literature.^22^ Compared to the study by Shayeb et al.^22^, which focused primarily on assay performance, our analysis offers additional insights by incorporating a broader tumor-type breakdown and evaluating the prevalence and co-occurrence of pathogenic ERBB2 mutations. These findings underscore the biological heterogeneity of ERBB2/HER2 alterations and may inform future biomarker-driven therapeutic strategies. Additionally, tumors with positive CN amplification were predominantly IHC3+ (75%), whereas intermediate amplification was more frequently associated with IHC2+ expression (47.8%). This correlation aligns with the established relationship between ERBB2 genomic amplification and HER2 phenotypic overexpression.^20^ Tumors with pathogenic ERBB2 mutations exhibited relatively low rates of HER2 IHC3+ expression (5%), underscoring the ERBB2/HER2 biology complexity and the need for deeper exploration into the functional impact of specific ERBB2 mutations. Additionally, intratumoral heterogeneity and temporal variations in HER2 expression have been documented, emphasizing the necessity for further research to refine IHC criteria and optimize patient selection for HER2-­targeted therapies.^23^ In the DESTINY-PanTumor02 study, HER2 expression was evaluated using gastric-specific criteria due to the lack of validated, disease-specific guidelines for other tumor types.^24^ Similarly, our study applied gastric scoring criteria for all cancer types except for breast cancer, where breast-specific guidelines were utilized. This standardized approach facilitated accurate comparisons and contributed to a comprehensive understanding of HER2 expression patterns across different malignancies.

Our findings also highlight the importance of carefully interpreting next-generation sequencing (NGS) results in HER2-targeted therapy. While ERBB2 mutations may indicate therapeutic targets, they do not always correlate with HER2 protein overexpression and should not determine treatment eligibility alone. In contrast, ERBB2 gene amplification, detected via NGS, strongly correlates with HER2 overexpression, warranting IHC confirmation. As IHC is the clinical gold standard, HER2 overexpression (IHC3+) should guide treatment decisions, including the use of T-DXd. Integrating NGS and IHC ensures appropriate HER2-targeted therapy selection, optimizing patient outcomes.

ERBB2 CNV amplification was observed in 6.6% of cases (3.1% positive, 3.5% intermediate), with pathogenic mutations detected in another 3.1%, primarily S310F, V842I, L755S, and R678Q—none of which overlapped with CNV events. This highlights the need for integrated ERBB2/HER2 testing (IHC, CNV, and mutation analysis) to capture distinct, clinically ­relevant alterations. Notably, these mutations can sustain oncogenic signaling and confer resistance to EGFR-targeted therapies, while ERBB2 amplification is linked to a non-inflammatory tumor microenvironment that may impact immunotherapy response.^25^^,^^26^

Recent advances in HER2-targeted therapies include monoclonal antibodies, ADCs, TKIs, bispecific T-cell engagers, and cell therapies. Among these, T-DXd has shown broad antitumor activity due to its high drug-to-antibody ratio (8:1) and bystander effect, earning FDA approval for HER2-positive and HER2-low breast cancer, ERBB2-mutant NSCLC, and HER2-positive gastric cancers.^16^^,^^27–31^ Data from DESTINY trials demonstrate that HER2 overexpression (IHC3+) remains the strongest predictor of response, while ERBB2 mutations show variable sensitivity and may inform future tumor-agnostic indications.^24^^,^^32^

The DESTINY-PanTumor02 trial evaluated T-DXd in patients with advanced or metastatic HER2-expressing solid tumors undergoing prior treatments. The trial demonstrated durable responses across various cancer types (ORR of 37.1%), particularly among patients with high HER2 expression (IHC3+) (ORR of 61.3%).^16^ This is the first trial to demonstrate sustained responses to HER2-targeted therapy in patients selected based on somatic ERBB2 mutations, regardless of tumor type.^16^^,^^23^^,^^24^ The phase III DESTINY-Breast04 (NCT03734029) and DESTINY-Breast06 (NCT04494425) trials demonstrated a significant improvement in PFS and OS in patients with previously treated HER2-low metastatic breast cancer, irrespective of HER2 expression levels (IHC1+ or 2+) or hormone receptor status.^33^^,^^34^ These findings led to FDA approval for this indication^23^^,^^34^ and have expanded the scope of HER2-targeted therapies.

In our dataset, HER2 IHC3+ expression was relatively rare, observed in only 3.1% of cases. This low prevalence is consistent with the need for more robust ERBB2/HER2 testing strategies, particularly in tumor types where HER2 overexpression is less common. However, the findings from DESTINY-Breast04 and DESTINY-Breast06 underscore that patients with HER2-low expression (IHC1+ or 2+) or ultra-low (any HER2 expression) can also benefit from T-DXd therapy^33^^,^^34^ In our cohort, the combined prevalence of HER2 IHC1+ and 2+ expression was 33% (13.2% IHC2+ and 19.8% IHC1+), suggesting a substantial proportion of patients who may still qualify for HER2-targeted therapies under these expanded criteria. This demonstrates that HER2-low testing, beyond IHC3+, can broaden the pool of eligible patients for treatment with novel agents like T-DXd. Furthermore, recent advances in ctDNA-based testing have shown promise in identifying HER2-amplified tumors, which may further enhance the clinical utility of HER2-targeted therapies.^35^ A multi-center Phase II Basket Trial demonstrated that ctDNA-based ERBB2 amplification detection could identify patients who benefit from T-DXd, especially where tissue biopsies are not feasible.^35^

Emerging modalities also show significant promise. Along with trastuzumab, other monoclonal antibodies, including pertuzumab and margetuximab, have shown efficacy in different tumor types as a monotherapy and combinations.^23^ Chimeric antigen receptor T cell (CAR-T) therapy targeting HER2 has demonstrated potential in preclinical studies for HER2-positive metastatic colorectal cancer (mCRC).^36^ Zanidatamab (ZW25), a bispecific antibody, simultaneously binds to 2 HER2 epitopes: ECD4, the trastuzumab binding domain, and ECD2, the pertuzumab binding domain was recently approved for the treatment of previously treated, unresectable, or metastatic HER2-positive (IHC3+) biliary tract cancer (BTC), based on findings from the HERIZON-BTC-01 trial (NCT04466891). In conjunction with this approval, the FDA also approved the VENTANA PATHWAY anti-HER-2/neu (4B5) Rabbit Monoclonal Primary Antibody as a companion diagnostic device to identify BTC patients eligible for treatment with Zanidatamab. In this context, evaluation of ERBB2 (HER2) alterations is clinically relevant, not only for well-known HER2-driven malignancies like breast or gastric cancer but also for all solid malignancies, as we showed in our cohort analysis.

On this evolving therapeutic landscape, a vital strength of the study lies in its systematic analysis of HER2 alterations, including IHC expression, CN amplification, and mutational patterns, providing a detailed understanding of HER2-driven oncogenesis and its potential therapeutic implications. However, study limitations should be acknowledged. As a single-center study, we have a relatively small sample size, and the findings may not be generalizable to broader populations. The retrospective design introduces potential selection bias. The limited representation of rare cancers, such as salivary gland and pleural mesothelioma, restricts statistical power for these subtypes. The samples analyzed were either core biopsies or surgical specimens. Given the reported intratumoral heterogeneity in HER2 expression, this variability may influence the correlation between copy number alterations and IHC expression levels. Furthermore, patient numbers with breast cancer were small in our study. Additionally, given that the PATHWAY anti-HER2/neu (4B5) assay is primarily to detect HER2 overexpression associated with ERBB2 amplification, it may lack the sensitivity to reliably identify low HER2 expression levels; particularly in light of emerging data from DESTINY-Breast04, highlighting the clinical relevance of low HER2 expression. The study also lacked longitudinal clinical data; however, these efforts are ongoing. Despite these limitations, this study sets a foundation for future research, emphasizing the need for prospective, multi-institutional studies with standardized testing protocols, functional validation, and clinical outcome integration to optimize the application of HER2-targeted therapies.

## Conclusion

This study comprehensively evaluates ERBB2/HER2 expression, amplification, and mutations across a diverse range of solid tumor types, offering valuable insights into the heterogeneity and clinical significance of ERBB2/HER2 alterations. The strong correlation between ERBB2 CN amplification and HER2 IHC expression underscores the importance of integrating genomic and phenotypic data to improve diagnostic accuracy and therapeutic decision-making. Among cases with ERBB2 mutations, only one exhibited positive CN amplification, and 2 showed intermediate CN amplification, further emphasizing these alterations’ distinct and independent nature. This finding highlights the complexity of HER2 biology and the necessity for comprehensive testing strategies encompassing IHC, CNV, and mutational analyses. While ERBB2/HER2 aberrations are well-characterized in breast and gastric cancers, our findings highlight the prevalence and potential clinical relevance of ERBB2/HER2 alterations in underrepresented cancer types, such as salivary glands, uterine, and cholangiocarcinomas. Furthermore, this study underscores the importance of identifying patients who may benefit from novel HER2-targeted therapies, such as T-DXd, by implementing comprehensive and routine ERBB2/HER2 testing, even in tumor types where such approval is not standard practice.

## Data Availability

All information is included in Table 1 in the manuscript.
