# Correction to: HER2 alterations across solid tumors: implications for comprehensive testing

**DOI:** 10.1093/oncolo/oyaf405

**Published:** 2025-12-23

This is a correction to: Ahmed Ismail, Chinmay T Jani, Nusrat Jahan, Malla Midhun, Arnab Basu, Garima Gupta, Bassel El-Rayes, Sejong Bae, Tyler Mattox, Cyntanna Hawkins, Rebecca C Arend, Mehmet Akce, Yanis Boumber, Aakash Desai, HER2 alterations across solid tumors: implications for comprehensive testing, *The Oncologist*, Volume 30, Issue 9, September 2025, https://doi.org/10.1093/oncolo/oyaf258

The name of the second author has been corrected in the article to read: “Chinmay T Jani”.

